# Formation in Interprofessional Education in Nursing and Medical Students Globally. Scoping review

**DOI:** 10.17533/udea.iee.v38n2e06

**Published:** 2020-07-10

**Authors:** Lylian Macías Inzunza, Víctor Rocco Montenegro, Jennifer Rojas Reyes, Marcela Baeza Contreras, Carolina Arévalo Valenzuela, Viviana Munilla González

**Affiliations:** 1 Nurse, Masters, PhD candidate. Assistant Professor, Universidad de Santiago de Chile. Chile. Email: lylian.macias@usach.cl Universidad de Santiago de Chile Universidad de Santiago de Chile Chile lylian.macias@usach.cl; 2 Physician, Specialist, Masters. Assistant Professor, Universidad de Santiago de Chile. Chile. Email: victor.rocco@usach.cl Universidad de Santiago de Chile Universidad de Santiago de Chile. Chile victor.rocco@usach.cl; 3 Nurse, Masters, PhD candidate. Universidad Nacional de Colombia. Colombia. Email: jrojasre@unal.edu.co Universidad Nacional de Colombia Universidad Nacional de Colombia Colombia jrojasre@unal.edu.co; 4 Nurse, Masters. Assistant Professor, Universidad de Santiago de Chile. Chile Email: marcela.baeza@usach.cl Universidad de Santiago de Chile Universidad de Santiago de Chile. Chile marcela.baeza@usach.cl; 5 Nurse, Masters. Professor, Universidad de Santiago de Chile. Chile. Email: carolina.arevalo@usach.cl Universidad de Santiago de Chile Universidad de Santiago de Chile Chile carolina.arevalo@usach.cl; 6 Nurse, Masters Student. Professor, Universidad de Santiago de Chile. Chile. Email: viviana.munilla@usach.cl Universidad de Santiago de Chile Universidad de Santiago de Chile Chile viviana.munilla@usach.cl

**Keywords:** interprofessional education, education, nursing, education, medical, students, nursing, medical students, medical, curriculum, review., educación interprofesional, educación en enfermería, educación médica, estudiantes de enfermería, estudiantes de medicina, curriculum, revisión., educação interprofissional, educação em enfermagem, educação médica, estudantes de enfermagem, estudantes de medicina, currículo, revisão.

## Abstract

**Objective.:**

This work sought to know the state of the art related to the theme of Interprofessional Education (IPE) in the training of Nursing and Medical students and the level of evidence developed thus far.

**Methods.:**

This was an exploratory systematic review, declared as scoping review by the Joanna Briggs Institute, JBI, in which a search was performed in Embase, Science direct, Pubmed-Medline, Academic search complete, BVS, Scopus and ERIC databases, limiting between 2009 - 2019 by using the DeCS and MeSH terms of Interprofessional education, education research, healthcare professionals, nursing and medicine, selecting 39 original articles after carrying out the review process with the criteria by the JBI.

**Results.:**

Four thematic nuclei emerged: Experiences and perceptions of interprofessional learning, Didactics related with IPE, Empirical indicators related with IPE, and Development of professional skills. The highest level of evidence is presented by the articles dealing with didactics; on the contrary, no articles were found that dealt with topics related with early inclusion of IPE in the medical and nursing curricula, which are currently necessary to complement the focus of patient-centered care.

**Conclusion.:**

The thematic nuclei show that the level of evidence in the literature is varied, although mostly descriptive in scope, highlighting the development of professional skills as a result of interprofessional education.

## Resumen

**Objetivo.:**

Conocer el estado del arte, relacionado con la temática de Educación Interprofesional (EIP) en la formación de estudiantes de enfermería y medicina, y el nivel de evidencia desarrollado hasta el momento.

**Métodos.:**

Revisión Sistemática exploratoria, declarada como *Scoping review* por la Joanna Briggs Institute -JBI-, en la que se realizó una búsqueda en bases de datos de Embase, Science direct, Pubmed-Medline, Academic search complete, BVS, Scopus y ERIC, entre 2009-2019 utilizando los términos DeCS y MeSH de *Interprofessional education, education research, healthcare professionals, nursing* y *medicine*. Se seleccionaron 39 artículos originales luego del proceso de crítica con los criterios de JBI.

**Resultados.:**

Emergieron cuatro núcleos temáticos: Experiencias y percepciones del aprendizaje interprofesional, Didácticas vinculadas a la EIP, Indicadores empíricos relacionados con la EIP y Desarrollo de competencias profesionales. El mayor nivel de evidencia lo presentan los artículos sobre empleo de didácticas; por el contrario, no se encontraron artículos que trataran temáticas relacionadas con la inclusión temprana de EIP en los currículos de enfermería y medicina, pues son actualmente necesarios para complementar el enfoque de atención centrada en el paciente.

**Conclusión.:**

Los núcleos temáticos muestran que el nivel de evidencia de la literatura es variado, aunque en su mayoría de alcance descriptivo, lo que resalta el desarrollo de competencias profesionales como resultado de la educación interprofesional.

## Resumo

**Objetivo.:**

Conhecer o estado da arte, relacionado com a temática de Educação Interprofissional (EIP) na formação de estudantes de enfermagem e medicina, e o nível de evidência desenvolvido até o momento.

**Métodos.:**

Revisão Sistemática exploratória, declarada como Scoping review pela Joanna Briggs Institute -JBI-, na qual se realizou uma busca em bases de dados de Embase, Science direct, Pubmed-Medline, Academic search complete, BVS, Scopus e ERIC, limitando entre 2009-2019 utilizando os termos DeCS e MeSH de *Interprofessional education, education research, healthcare professionals, nursing and medicine*. Se selecionaram 39 artigos originais após de realizado o processo de crítica com os critérios de JBI.

**Resultados.:**

Emergiram quatro núcleos temáticos: Experiências e percepções da aprendizagem interprofissional, Didáticas vinculadas à EIP, Indicadores empíricos relacionados com a EIP e Desenvolvimento de competências profissionais. O maior nível de evidência o apresentam os artigos sobre emprego de didáticas; pelo contrário, não se encontraram artigos que trataram temáticas relacionadas com a inclusão precoce de EIP nos currículos de enfermagem e medicina, sendo atualmente necessários para complementar o enfoque de atenção centrada no paciente.

**Conclusão.:**

Os núcleos temáticos mostram que o nível de evidência da literatura é variado, embora na sua maioria de alcance descritivo, ressaltando o desenvolvimento de competências profissionais como resultado da educação interprofissional.

## Introduction

One of the ways to respond to the *Millennium Development Goals* with respect to universal health is related with guaranteeing the formation of competent professionals, who are focused on responding to the needs of the people through collaborative work among the different health disciplines; however, this conception tends to be removed from reality in global health systems, where confrontations among professionals play a harmful role for users. This is how interprofessional education (IPE) emerges as a response to this problem, as a teaching and learning approach that joins students from two or more professions to learn about their roles,(1,2) in which they learn with, learn of, and learn about each other to improve collaboration and the quality of health care.(3)

As presented, one of the principal problems to which interprofessional education responds is the definition of roles among health professionals, especially between physicians and nurses; this has been considered a constant problem in the clinical setting in which a thin line exists between what each one can do and can cause confusion in the roles. According with the Theory of Roles,(4) the role is a set of prescriptions that define the behavior of a group member in a given position within the group; however, when students are not in contact during their formation with other disciplines with which they will work, it is difficult for them to manage to identify the extent of their tasks or define which these are.

According with a systematic review by Reeves *et al.,(*5) IPE seeks to improve collaboration among distinct types of health professionals and from social care, premise that is based on 15 studies reviewed, which assessed the effectiveness of IPE interventions, evidencing positive results regarding patient satisfaction, conduct of the collaborating team, and diminished rates of clinical errors in the service teams. The evidence from the reviews found(5,6) are aimed exclusively at the impact of interventions, added to their focus being given within the context of collaborative practices, making it necessary to inquire if during the period of university formation, and within the curricula of Nursing and Medical programs, these incorporate strategies to promote IPE.(7) Consequently, the review proposed develops more broadly the topic of this theme, not only focusing on interventions but on the experiences provided by this education and the skills that can be improved with it. 

To develop IPE, we must consider methodologies that permit students to be active, interactive, reflexive, and be centered on the patient. These methods may be used to create opportunities, to compare and contrast the functions and responsibilities, power and authority, ethics and codes of practices, knowledge and skills; to establish effective relations and develop and reinforce aptitudes for collaborative practice.(8,9) Having described its importance in professional formation, it is imperative to know the state of the art related with the theme of interprofessional education (IPE) on the formation of students from the Nursing and Medical careers and the level of evidence developed until now, thus, determining the contributions and knowledge gaps present in the literature. 

## Methods

Descriptive Scoping Review(10) which sought to answer the questions: What is the current state of scientific knowledge of the IPE phenomenon in Nursing and Medicine? What is the evidence of its applicability, benefits, and limitations? What are the research gaps on this theme? The search was performed in the Embase, Science direct, Pubmed, Medline, Academic search complete, BVS, Scopus, and ERIC databases in English, Spanish, and Portuguese, limited to between 2009 and 2019, with this time range being adequate to encompass the evolution and current status of the theme, which is relatively novel in Latin America. The study used DeCS and MeSH terms of interprofessional education, education research, healthcare professionals, nursing and medicine, performing the search with equations using AND and OR Boolean operators. Although the context of this review is found globally, for effects of the search, in some of the databases the information was filtered by Latin America, North America, Europe, Asia, Africa and Oceania, which permits recognizing the places in the world where the IPE theme is more or less developed.

**Figure 1:**
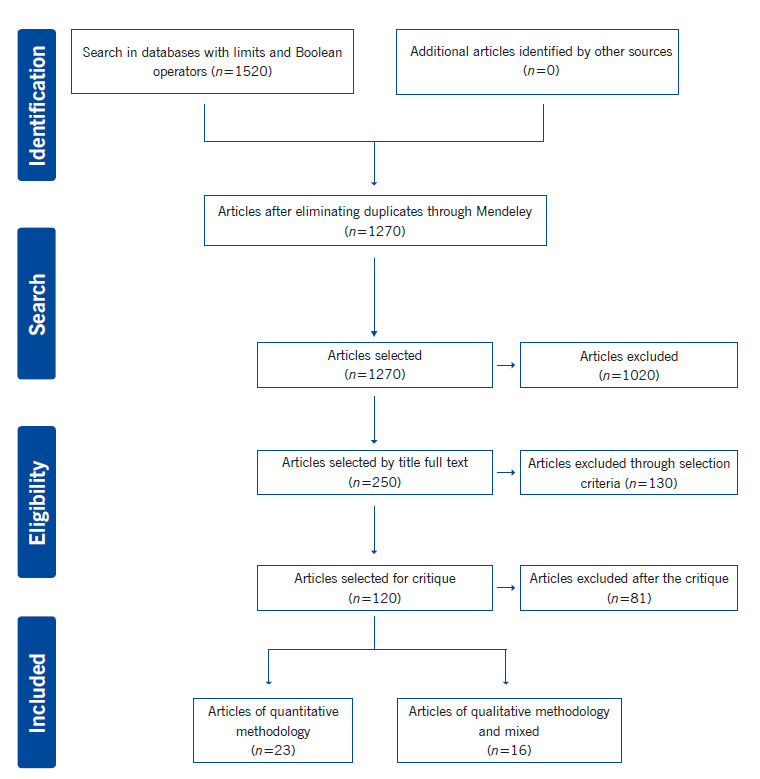
Flow diagram of the article search and selection strategy

The inclusion criteria for this review considered original articles, which present data from research results available in full text, which included subjects like professors and/or Nursing and Medical students. The study excluded texts and theses due to their length and because they address the theme of education without being specific on the interprofessional. It also excluded reflexion articles because they do not contribute concrete and specific results, which could be classified within the levels of evidence provided by the JBI(11) and review articles because contribute secondary sources of information and contain interpretations by the authors about the primary sources, which for this review, are not relevant. The search and selection strategy of articles is detailed in Figure 1, which has been adapted from the PRISMA flowchart for Scoping Review.(12)

For information analysis, the articles were collected in the Microsoft Excel program, where a matrix was created with their critique and analysis. To conduct the critique of the quality of the articles, the rigor criteria were used in the research, bearing in mind their approach. For qualitative studies, the review applied criteria by Guba(13) of credibility, transferability, dependence, and confirmability. Quantitative studies used criteria of internal, external, construct, and statistical validity.(14)

Regarding the analysis of theme addressed in the articles, development of the matrix defined as variables to consider the research approach, study design, data collection manner, analysis used, their principal results, and the conclusions; then, with this information condensed, the work followed the steps exposed by Peters *et al*.,(10) which include question, objective, inclusion criteria, participants, principal concept, context, search and selection of articles, extraction and classification of results and discussion.

## Results

The bibliographic search led to the selection of 120 original articles, which after the critique excluded 81 of them, 45 quantitative studies which mostly had faults in their internal validity; 25 qualitative studies that did not clarify the confirmability of the data or the reflexivity in the research process and 11 mixed studies due to declaring this methodology without evidencing its integration in the results. In the end, 39 original articles were analyzed, having as criteria their thematic and/or methodological affinity, thereby, deriving into four thematic groups: Experiences and perceptions of interprofessional learning, Didactics linked to IPE, Empirical indicators related with IPE, and Development of professional skills. With respect to the methodological distribution of the articles after their integration in the principal concepts, Table 1 shows that the concept most approached was the development of professional skills by 36%. The predominant methodological approach was qualitative research by 28.2% of the total analyzed and development of psychometric research with 12.8%. 

**Table 1:** Distribution of 39 articles reviewed according to the methodology employed and the principal concept approached

Concepts	Experimental methodological approach	Experimental methodological approach	Experimental methodological approach	Experimental methodological approach	Experimental methodological approach	Total *n* (%)
Concepts	Qualitative *n* (%)	Non-experimental *n* (%)	Quantitative *n* (%)	Psychometric *n* (%)	Mixed *n* (%)	Total *n* (%)
Development of professional skills	3 (7.7)	4 (10.3)	4 (10.3)	0	3 (7.7)	14 (35.9)
Didactics linked to IPE	2 (5.1)	2 (5.1)	6 (15.4)	0	1 (2.6)	11 (28.2)
Experiences and perceptions of interprofessional learning	6 (15.4)	2 (5.1)	0	0	1 (2.6)	9 (23.1)
Empirical indicators related with IPE	0	0	0	5 (12.8)	0	5 (12.8)
Total	11 (28.2)	8 (20.5)	10 (25.6)	5 (12.8)	5 (12.8)	39 (100)

With respect to their geographic location, 61.5% of the articles are from North America, comprising the United States and Canada. Two articles were identified in Latin America and none in Africa. Table 2 shows, not only how limited IPE research is in Latin America, but the progress in design and implementation of interprofessional formation programs in Nursing and Medicine taking place in the United States and Europe.

**Table 2:** Articles located by geographic location

Location	Countries reporting	Number (%)
North America	The United States (*n* = 21) and Canada (*n* = 3)	24 (61.5)
Latin America	Chile (*n* = 2)	2 (5.1)
Europe	Spain (*n* = 3), United Kingdom (*n* = 1), Germany (*n* = 2), Serbia (*n* = 1), Bosnia and Herzegovina (*n* = 1).	8 (20.5)
Asia	Japan (*n* = 1), Lebanon (*n* = 1), and Saudi Arabia (*n* = 1)	3 (7.7)
Oceania	Australia (*n* = 2)	2 (5.1)

Besides the contributions found in each of the articles reviewed, which will be presented in the discussion, the work presents the levels of evidence according with the JBI(11) and the research gaps according with each thematic category.

## Discussion

The following presents the discussion among the relevant findings of the emerging themes in this review, levels of evidence, and explicit gaps. 

### Development of professional skills (n=14)

Contributions found

Research on IPE has demonstrated that it favors the development of professional skills, framed within knowledge, skills, and attitudes that permit optimizing interpersonal relationships with colleagues, peers, and users, besides recognizing their shared and autonomous roles and of supporting the capacity to make shared decisions. Initially, on addressing the preparation of students upon IPE, Judge *et al.,(*15) proposed a quasi-experimental study to evaluate the response capacity against IPE through cases that had to be solved by students from odontology, medicine, nursing, pharmacy, dietetics, and physical therapy. These authors concluded that the average score in the Scale of preparation for interprofessional learning in nursing, after the intervention, was higher compared with other disciplines.

Now, on approaching the professional skills developed with IPE, we approach the perception of students and its measurement with valid instruments; thus, from this qualitative approach diverse authors gathered from the students that the knowledge and skills they developed most were related with the capacity for reflexion, communication, identification of their role and that of the other, construction of team relationships, collaboration, and mediation to make decisions, to provide patient-centered quality care.(16,17) Other studies have described that when students from the health sciences learn and work in interprofessional teams, they obtain greater clarity of their role and ability to plan tasks than when they are in intra-professional teams, added to the positive impact this has on the sensitization on the role of other professions.(18,19)

Moreover, the interventions conducted based on didactics around IPE evidenced promotion in the development of skills related with communication, teamwork, conflict resolution, decision making, roles and attitudes on care by the interprofessional team.(20-22) For Baker and Durham,(20) elaborating an IPE course permitted improving collaboration, conflict resolution, and communication among students from medicine, nursing, and pharmacy; aspects also indicated by Castillo Parra *et al.,*(23) emphasizing on the construction of new knowledge with collective learning. Other authors who used simulation for interprofessional learning described that their participants changed their preconceived ideas on physician, nursing, and pharmaceutical professionals.(21)

In a more practical level, a program was designed consisting of interviews of patients, followed by a team meeting to develop an integral care plan. Statistically, no significant changes were noted on the attitudes, but the comments from the focal groups highlighted the value of collaboration among health professions and the potential benefit of participating more than once to obtain longer lasting experience with a patient and have additional exposure to work as interprofessional team.(22) It is also highlighted that IPE on the care of individuals with chronic diseases, like diabetes, where physiopathological knowledge is transversal in medicine and nursing, permits development of clinical skills.(24) From the perspective of bioethics in the practice of health sciences, diverse studies found(25-27) managed to support that under IPE development is achieved of this vision of ethics and values in students added to the practice and interdisciplinary teamwork. Thereby, it is recognized that at curricular level the programs of health sciences have tried to wage on IPE as a way of enhancing professional collaboration, identifying the role to perform, and developing transversal values for the care of patients, in addition to the joint commitment to maintain knowledge updated to benefit the users, that is, application of evidence-based practice but interprofessional.(26) In this same sense, the study by Harper Boland *et al*.,(25) determined that with IPE students improved their skills for teamwork (*p* value of the pre and post difference <0.001) and their level of trust on themselves and on others, added to the students expressing greater comprehension of the differences in the values and ethics of the multiple professions, which generated increased recognition and respect for the differences within the work carried out by each profession. 

Finally, the study by González *et al*.,(28) evaluates the impact of IPE in developing skills inherent to this learning, such as communication, role definition, teamwork, and decision making, finding a positive effect in the students’ self-perceptions, especially with interprofessional communication, in the dimensions of oral expression, active listening, and conflict management (*p* = 0.018; *p* = 0.018; *p* = 0.036 and *p*(0.001, respectively), revealing that this type of education helps future health professionals to center on caring for people and not on the exclusivity of their roles. 

The evidence collected shows that the skills developed most were role definition and teamwork aimed at the care of patients being safe and humanized, supporting the idea that the implementation of IPE in undergraduate health sciences curricula prepares students for collaborative practice, becoming the opportunity to learn from each other; however, consensus must exist and the political will of the Faculties and Deanships to include the work with other professions within their study programs.

Levels of evidence

Of the articles analyzed in this thematic, six are in a level of significance of which three are in level 2(22,23,25) because they are studies of mixed methodology and the rest in level 3(16,17,26) because they are qualitative studies. With respect to the level of effectiveness, eight articles are presented, of which four correspond to level 2C(15,18,21,28) because of their quasi-experimental designs; one article in level 3E(24) due to being a study without control group and three articles with level 4B(19,20,27) because of being investigations with cross-sectional design.

Explicit gaps

Of the articles analyzed on this thematic, some gaps are derived that guide to research in professional skills within IPE, such as:

- Describe the development of professional skills framed within interpersonal aspects and of critical judgment in the practice.
- Identify the influence of collaborative work on the development of specific professional skills in care and community areas.
- Develop educational programs to promote learning of interpersonal skills and collaborative work.

### Didactics linked to IPE (n=11)

Contributions found

With respect to the concept of didactics, diverse results are noted on the description and development of pedagogic strategies; thus, predominating the use of standardized simulation and role play, which are evaluated positively, given that they permit acquiring skills to develop necessary skills to provide better care. Also, the use of other specific collaboration strategies with students and professionals in clinical settings, like using TeamStepps and Aspire model have permitted improving the quality and safety of care, strengthening health teams. Currently, evidence exists of new strategies, like using e-learning that has had favorable results. The active methodologies used in IPE generate a positive learning experience of soft skills perceived by students in health careers. They have reported that it helps them to develop skills to clarify roles, use democratic and horizontal models in decision making, and interprofessional communication based on respect/trust, generating greater awareness as a result of the interactive and dialogical nature of the didactics and by having an academic from communications.(29,30)

Interprofessional education in complex scenarios of high-fidelity simulation and problem-based learning with experience practice permits students to improve not only communication, but also the trust from patients in an interprofessional team, achieving that established in the study plans.(31,32) Among the basic recommendations to apply simulation, there is the suggestion of selecting adequate criteria for self-appraisal and coordination in administrative aspects; planning must include the development of teaching materials and supply of efficient technical equipment.(33) From a real professional scenario in clinical units, continuous training of the health staff is quite common, focused on generating quality and safety environments, and using diverse didactics. Two experiences, use of TeamStepps and ASPIRE model, experienced by professionals and graduate nursing students, medicine and other health careers, has evidenced positive results after sessions of interprofessional collaboration through workshops, discussions with experts, and simulations. The participants presented improvement in team structure, communication skills, leadership, situation monitoring, and mutual support.(9,34)

Among the strategies most often used in IPE, there is simulation; findings of the experiences with this type of didactic have resulted favorable. Upon evaluating the perception in the socialization and interprofessional assessment (91 students from nursing, medicine, and pharmacy), statistically significant improvements were evidenced; 92% enjoyed the interaction opportunity and 81% report that it sharpens their awareness of the roles other disciplines perform in the delivery of care.(35) Similarly, in another experience, 329 students from health professions, under the same simulation didactic, 90% was very much in agreement with the advantages and benefits of this and 60% stated that the sessions would change their professional behavior.(36) 

Another experience that evaluated communication skills, exchange of information, and interaction of teamwork with 166 students from health careers, through a pre- and post-test of the interprofessional simulation, evidenced no statistically significant differences, but the participants considered the experience valuable and declared that the observations by professors and standardized patients were very useful in their professional growth.(37) However, simulation is not foreign to some limitations and barriers in the experiences, having difficulties, like in the programming schedules, funding, and staffing.(36,37) Regarding ICT, IPE learning strategies were compared on knowledge, skills, and teamwork attitudes; for mixed learning (classroom plus e-learning) versus virtual learning (e-learning). Both groups reported significant increase in teamwork skills, but not in communication and conflict resolution; nevertheless, the mixed-learning cohort reported improvement in the domains of attitude, while the virtual-learning cohort reported improved leadership.(38)

Simulation is an excellent learning strategy because it provokes a positive impact by being a strategy that generates benefits in the development of soft skills. It is important to recognize the benefits offered by this didactic, which is why it may be worth to incorporate it to curricular plans in Nursing and Medicine careers. Other didactics that support and permit overcoming barriers must be considered, like using virtual sessions of which more research is warranted. 

Levels of evidence

As per the level of evidence in this thematic, eight articles have a level of effectiveness, represented by six quasi-experimental studies classified in level 2C(9,34,38) and two articles with cross-sectional design placing them in level 4B.(31,32) Regarding level of significance, there are three articles of which one is in level 2(33) due to its mixed methodology and the other two in level 3(29,30) for being qualitative studies.

Explicit gaps

The gaps derived from the analysis of articles about this theme are aimed at aspects of IPE, such as:

- Implement and test different didactic strategies that have been developed and evaluated as effective.
- Describe the experiences of professors and students on the use of didactics, like standardized simulation, role play, and e-learning in IPE.
- Evaluate the impact of implementing these didactics within formation curricula of health programs.

### Experiences and perceptions of interprofessional learning (n=9)

Contributions found

With respect to the experiences of students from diverse health professions regarding IPE, satisfaction exists on their participation in collaborative activities, added to the fact of knowing the emotional experience and roles of each one in the health team is important to achieve practices focused on teamwork. Additionally, these instances permit identifying benefits, facilitating aspects, as well as barriers and/or factors affecting their development.

In interprofessional learning, individuals cannot simply come together in situations to learn to work together, rapport must be developed through interpersonal skills, given that said relationship has the potential to break down some of the stereotypical perceptions professionals have among themselves, making it easier and natural for them to work together.(39) To achieve the aforementioned, recognition of emotions gains relevance; thus, interprofessional activities in diverse settings generated similar emotions in students, who considered that identifying blind spots of their own role and borrowing the co-worker’s lens to comprehend what they do and experience, promotes convergence into a shared vision on caring for the users.(40) 

Identifying the professional role becomes a central element as a result of IPE, given that by reflecting on their collaborative learning students recognize a change in preconceived authoritarian ideas about themselves and on the recognition of the roles of other professions.(41,42) However, students also reported that collaboration in IPE was seen as something secondary to principal learning.(43) From the aforementioned, the presence of aspects that affect the IPE can be deduced. In this regard, leadership, working together, and a common setting for work groups are recognized as facilitating aspects, achieving as benefits better care for the user; development of optimal interprofessional relationships, and work satisfaction. Learning was hindered by inadequate staffing, productivity pressure, and programming challenges, considering this last element the principal negative factor. Barriers included leadership with hierarchical approaches, poor communication, and lack of knowledge of roles.(44,45)

In an IPE experience with 704 medical and nursing students in primary health care (PHC), perception regarding interprofessional work and interdependence evidences that 98% consider that interprofessional work is important, with women being most critical on the level of importance on teamwork (*p* <0.01), along with being more demanding in considering it essential in PHC (*p* = 0.035).(46) In relation with the attitudes of educators with respect to an IPE experience, professors who had participated in IPE reported higher intention to participate or continue participating than professors without experience in such. The combination of the disposition perceived by administrators and attitudes toward IPE was the best predictor of the intention to participate in IPE, although no significant differences were detected among the groups of professors with respect to these attitudes.(47)

The importance of knowing these experiences and perceptions on IPE for the curricula of health programs implies incorporating these types of instances in Nursing and Medicine, given that student-centered educational programs may be favored by using IPE, where students are able to channel their emotions together, favoring their regulation among peers. Finally, today, IPE opens undoubtedly a gap that can be analyzed from the gender perspective, of how women visualize comprehensiveness and teamwork as a transcendental factor in their formation.

Levels of evidence

The level of evidence in this thematic is predominantly significant, with seven articles, of which one is in level 2(42) for being of mixed methodology and the other six in level 3(39,41,43,45) due to being qualitative studies. Only two articles had a level of effectiveness in 4B(46,47) due to being cross-sectional correlational descriptive studies.

Explicit gaps

For this theme, the articles have derived gaps focused on the perceptions and descriptions of the development of IPE, such as:

- Recognize and identify one’s and the other’s professional.
- Integrate IPE early in the curricula of health programs.
- Understand the positive and negative aspects of IPE as improvement opportunities.
- Know the perception of subjects of care on the effect or impact of IPE on health professionals.

### Empirical indicators related with IPE (n=5)

Contributions found

Empirical indicators refer to processes measuring the IPE phenomenon, hence, the measurement instruments will be described, considering that the literature reports that these have been aimed at evaluating preparation processes in interprofessional learning, interprofessional collaboration, attitudes toward IPE, and the results this can have on the practice. Beginning with the preparation Scale for interprofessional learning, Serbian authors made their process of adaptation and validation.(48) This instrument was designed in 1999 by Parsell and Bligh(49) to evaluate results expected from collaborative learning in medical students; it is comprised of three factors with 19 items measuring teamwork and collaboration, professional identity, and professional roles. For the Serbian adaptation and validation of this scale, it was determined that the exploratory factor analysis with 19 items revealed two factors representing 51.1% of the total variance with internal reliability α = 0.90.(48)

Moreover, Iverson *et al*.,(50) designed and validated an instrument to assess interprofessional learning and the results it can have on the practice. Thus, these authors, through the literature and a panel of experts identified four basic aspects to consider within the instrument: values and ethics; roles and responsibilities; interprofessional communication; and teamwork. From there, 26 items were conformed, evaluating the skill with dichotomy scale, which do not have construct validity, but in its face validity, the instrument had inter-evaluator scores ≥ 0.78 and content validity was = 0.93, considered acceptable. Retaking the thematic of ethics and professional values mentioned, a scale was designed to identify bioethical aspects shared by diverse deontological codes of the health professions. With the values identified, a Likert-type survey of attitudes was designed, which requested your giving value to your estimated ethics of each of the values in relation to your profession. Reliability was tested through Cronbach’s alpha, obtaining a score of 0.905; however, the authors do not report other statistical tests to validate this instrument.(51)

Continuing with that referring to the disposition for interprofessional learning, a 27-item instrument was created from the literature to measure attitudes toward interprofessional collaboration in students and health professionals, denominated Jefferson Scale of Attitudes toward Interprofessional Collaboration (JeffSATIC). From the factor analysis performed for the construct validity, two factors emerge: working relationships and responsibility, which through Bartlett’s sphericity test represents 51% of the total variance; against confidentiality, Cronbach’s alpha was 0.80.(52)

Lastly, an instrument was identified to measure skills derived from interprofessional education; this was developed by authors from the United States to evaluate results related with the collaborative practice at undergraduate level. Based on a document published on the theme, they created a 42-item questionnaire applied to 481 participants for its validation. Four factors were defined: teamwork; values and ethics; interprofessional communication; and roles and responsibilities, thus, the factor analysis demonstrated that the instrument explains 79% of the variance. Each component showed a high degree of internal consistency with Cronbach’s alpha ranging from 0.96 to 0.98.(53)

From these empirical indicators described, it may be concluded that greater progress is still needed to measure aspects or dimensions related with IPE, especially on the thematic of professional skills shared by health professionals, added to patients’ perceptions of care from interdisciplinary teams. The instruments described are tools that must be adapted and validated to the context so they can be used in suitable manner.

Levels of evidence

The construction and validation of scales and instruments represent the progress of knowledge toward empirical indicators that permit measuring the IPE phenomenon in concrete and tangible manner; however, due to the psychometric approach, where validity and reliability results are obtained, no evidence is produced as such that should be classified in the JBI levels.

Explicit gaps

For this theme, the research gap is more methodological tan thematic, which is why it is necessary to conduct psychometric research that construct and validate instruments that measure skills in integral and scale manner regarding skills, knowledge, attitudes, and organizational environment to measure the impact of IPE on health professionals. 

Finally, knowing the role of another health professional facilitates performance in relation with the limits of action and that influences undoubtedly on the leadership future nursing or medicine professionals will assume in a given situation, where recognition of what others do favors communication; thus, IPE experiences are an opportunity for students to learn. It is suggested to include this methodology in the Nursing and Medical curricula if a true effect is expected on the change of stereotypes from the other professions. 

Among the limitations of this review, we have that due to this being an exploratory review, it was not possible to fully guarantee that it was systematic and was not focused on a methodology of specific studies, added to the selection bias, which excluded articles beyond the range of time established for the search and documents, like theses or degree works that could have contributed more information to the IPE thematic. 

It can be concluded that the state of the art related with the interprofessional theme on the formation of nursing and medical students globally proved varied, although most of the productions were of descriptive scope. Four thematic groups are highlighted therein: Experiences and perceptions del interprofessional learning, Didactics linked to IPE, Empirical indicators related with IPE, and Development of professional skills, with the thematic of didactics having the greatest progress, with presence of experimental studies that demonstrated the effectiveness of simulation activities and e-learning to develop knowledge and skills that lead to interprofessional learning, with these coming mostly from North America and Europe, where the highest scientific production has been reached in this theme.

Research gaps primarily concentrate in demonstrating the importance of early integration of IPE in the curricular syllabus of the Nursing and Medicine careers; evaluating the different types of IPE didactics within the formation curricula of health programs and identifying the influence of collaborative work on the development of professional skills, especially interpersonal skills. It is relevant to know the perception of patients on the impact of IPE on their care and identify the influence of collaborative work on the development of professional skills, as well as on the perception of the faculty body involved in teaching interpersonal skills through interprofessional education.
